# Effect of the Air Filtration System Replacement on Embryo Quality in the Assisted Reproduction Laboratory

**DOI:** 10.1055/s-0038-1670715

**Published:** 2018-10

**Authors:** Karine Queiroz Poletto, Yanna Andressa Ramos de Lima, Mário Silva Approbato

**Affiliations:** 1 Faculty of Medicine, Centro Universitário UNIRG, Gurupi, TO, Brazil; 2 Center for Human Reproduction, Department of Gynecology and Obstetrics, Universidade Federal de Goiás, Goiânia, GO, Brazil

**Keywords:** controlled environment, air quality, in vitro fertilization, embryo, volatile organic compounds, ambiente controlado, qualidade do ar, fertilização in vitro, embrião, compostos orgânicos voláteis

## Abstract

Improving infrastructural conditions of the in vitro fertilization laboratory, such as the air quality, has profound positive effects on embryo culture. Poor environmental conditions reduce the rate of embryo formation and, therefore, of pregnancy. This review article presents important publications regarding the impact of air quality in human reproduction laboratories on embryo quality, pregnancy success, and live births. The studies demonstrate that the replacing the air filtration system improves significantly the environmental air quality, and, consequently, improves laboratory parameters, such as the fertilization rate, the number of blastocysts, the embryo implantation rate, and the number of live births. On the other hand, improving air quality decreases the number of abortions. Therefore, environmental parameters that improve embryo quality and increase healthy child birth rates must be the main targets for the assisted reproduction laboratory quality control.

## Resumo

Melhorar as condições de infraestrutura do laboratório de fertilização in vitro, com influência na qualidade do ar, tem efeitos positivos profundos na qualidade do embrião. As más condições ambientais do ar reduzem a taxa de sucesso na formação de embriões e a taxa de gravidez. Este artigo de revisão apresenta importantes publicações sobre o impacto da qualidade do ar dentro do laboratório de reprodução humana na qualidade do embrião, no sucesso de gravidez e no número de nascidos vivos. Os estudos demonstram que a troca do sistema de filtração de ar melhora significativamente a qualidade do ar ambiente, e consequentemente, melhora os parâmetros laboratoriais, tais como a taxa de fertilização, o número de blastocistos, a taxa de implantação e o número de nascidos vivos. Por outro lado, a melhora da qualidade do ar diminui o número de abortos. Portanto, os parâmetros ambientais que melhoram a qualidade do embrião e aumentam as taxas de nascimentos de crianças saudáveis devem ser os principais alvos para o controle de qualidade do laboratório de reprodução assistida.

## Introduction

Infertility is a common disease, which drives several couples to seek treatment with assisted reproduction techniques. According to surveillance studies, 15% of couples around the world are infertile, and 85% of them can be treated with assisted reproduction.^1^

Much progress has been made in the last two decades regarding reproductive medicine in the treatment of many types of infertility. The population of patients who need this type of treatment is composed of several groups from different socioeconomic and cultural statuses. Human assisted reproduction consists of highly complex techniques that aim towards the efficient and safe handling of gametes, to produce viable embryos that lead to the birth of healthy babies, safeguarding the health of individuals involved in the process.^2^ Many factors contribute to the efficiency of assisted reproductive techniques, such as the laboratory environment and air quality.^3^ In fact, air quality control within the in vitro fertilization (IVF) laboratory is one of the major determinants of the assisted reproduction success, as it was shown to significantly increase parameters such as live birth rates.^4^ ^5^ Therefore, the implementation of a quality management system is crucial to achieve and maintain optimal and safe conditions in the biological material handling process.^2^

Poor air quality in the IVF laboratory is a known risk factor in the culture of gametes and human embryos. Hence, analytical methods have been used to identify, measure and select air pollutants to assess the risk they pose to the IVF system. However, concentrations of air contaminants that jeopardize gamete quality or cause embryo toxicity are poorly defined. Targeting the negative effects of poor air quality requires an understanding of how potentially toxic substances can infiltrate the laboratory, the equipment and the culture media. Further understanding of site-specific air quality may lead to a better consideration of laboratory design and management strategies that might minimize the deleterious effects of air contamination on early in vitro embryo development.^6^

The present review article presents important publications regarding the impact of air quality within human reproduction laboratories on embryo quality, pregnancy success and live birth rates.

## Methods

The articles used in this review were retrieved from several databases (Bireme, PubMed, Medline) and directly from human reproduction journals. The following search terms were used: *clean rooms,* *cleanroom*, *air quality*, *embryo quality*, *volatile organic compounds*, and *embryo culture*. In order to be included in the review, the studies had to be about: infrastructure and quality control at the IVF laboratory; microbiological monitoring and control in clean rooms; pollutants present in clinical and laboratory environments; air contaminants and the impact on embryo quality and pregnancy success; and air filtering systems in clean rooms. We excluded studies that did not approach quality control in the laboratory or impacts of the environment air on embryo quality. More than 1,200 articles were evaluated, and 45 fulfilled the inclusion criteria.

## Results

### Quality Control at the Human IVF Laboratory in Brazil

Infrastructure and quality control in the IVF laboratory, which are the primary determinant of clinical success, might be judiciously observed by embryologists and laboratory directors to meet regulatory standards requirements.^7^ To prevent the transmission of infectious diseases via biological samples, several countries have created legal regulation criteria for an adequate functioning of assisted reproduction centers. The European Union,^8^ the United States,^9^ Australia, and New Zealand have tightly standardized recommendations for “good practices” at the IVF laboratory. Similarly, the Brazilian government has recently established a normative resolution that regulates the quality control for assisted reproduction centers (Resolution of the Collegiate Board of Directors [RDC, in the Portuguese acronym] no. 33 [RDC33], of February 17, 2006).^10^

The main objectives of RDC33 are: to guarantee technical and quality standards throughout the process of obtaining, transporting, processing, storing, releasing, distributing, registering, and using germline cells and tissues for therapeutic purposes; to ensure the availability of germline cells and tissues from voluntary and anonymous donations for therapeutic purposes by third parties, or to maintain the reproductive capacity of the donor, with quality and safety; and to regulate the functioning of germline cell and tissue banks for reproductive therapeutic purposes.

The Brazilian National Health Surveillance Agency (Anvisa, in the Portuguese acronym) established, since the promulgation of the resolution, a one-year period for the denominated Cells and Germinative Tissues Banks (BCTG, in the Portuguese acronym) to adapt to the resolution.^10^ Among the several parameters required, the IVF laboratory must install an air conditioning system identical to the one within the sample processing room. Aditionally, the handling of samples should only be performed in a clean area classified at least as ISO 5, according to the regulation NBR/ISO 14644–1 of the Brazilian Association of Technical Standards (ABNT, in the Portuguese acronym). By definition, a Clean Room Class 100/ISO 5 is a standardized place in which the particle concentration (< 0.5 μm) is not higher than 100 particles per cubic foot or 3,520 particles per cubic meter. The existence of an environment classified as 100/ISO 5 is required in the micromanipulation environment of gametes and embryos, such as the unidirectional flow cabin. The Brazilian standard laws require that the filtration system include activated carbon filters capable of removing particles and volatile organic compounds (VOCs).^10^

The RDC33 was updated, revoked and recently replaced by RDC23 (from May 27, 2011),^11^ which provides technical regulation for the BCTG operations and practices. In its 53rd article, it is stated that the BCTG must perform a microbiological control of environments and equipments (such as CO_2_ incubators) used to process cells, germline tissues and embryos. These analyses should be performed every six months or less, according to the validated protocols of each BCTG.^11^

Human reproduction laboratories should contain a positive pressure room, particulate air filters, asepsis and decontamination care. The environment where the micromanipulation of gametes is performed must not have any hydro-sanitary installation, such as sinks or drains. The air conditioning system should maintain a positive pressure in relation to adjacent environments, temperature control between 23 and 27°C, relative air humidity between 40 and 70%, minimum total airflow of 45 (m^3^/h)/m^2^, minimum outdoor airflow of 15 (m^3^/h)/m^2^, and minimum air filtering with G3+ activated carbon filters +F8. Air filters of the high efficiency particulate air (HEPA) type and activated carbon for volatile organic substances should be used.^11^ HEPA or ultra-low particulate air (ULPA) filters are the final filtration elements, since they present high efficiency in the removal of submicron particles. The efficiency of these systems in reducing microbiological contamination was previously validated.^12^ ^13^

Microbiological monitoring in clean rooms is part of the quality assurance control routine: the objective is to systematically measure and evaluate the amount of live microorganisms present in these environments and to guide preventive and corrective measures to eliminate possible sites of contamination.^14^ These monitoring practices within controlled environments are also useful to evaluate the effectiveness of cleaning and sanitation procedures. However, regardless of the degree of complexity, this monitoring is not able to identify and quantify all the microbial contaminants present in these controlled environments. Nevertheless, routine monitoring should provide sufficient information to determine that the controlled environment is operating within a suitable condition.^15^

Although the use of an efficient air system and microbiological monitoring have been demonstrated as crucial measures to improve environmental conditions, embryologists and staff of the IVF clinics also play an important role in creating a clean and safe environment. Embryologists should be aware of their responsibilities and should be well trained to avoid and solve contamination problems.

### Air Contaminants and the Impact on Embryo Quality and Pregnancy Success

Several studies have demonstrated that gametes and human embryos are sensitive to the pollutants present in clinical and laboratory environments.^16^ ^17^ ^18^ ^19^ ^20^ ^21^ ^22^ ^23^ Atmospheric agents considered potentially toxic to human embryos include particles (smoke and dust), VOCs (alcohol and acetone), inorganic gases (carbon monoxide), components associated with laboratory facilities (adhesives, paint, cleaning products), among others.

A previous study demonstrated that laboratories that manipulate human gametes and perform embryo culture have sources of air contamination that exceed the levels measured in domestic, commercial and school environments.^24^ Compressed gas, cleaning and sterilizing agents, plastic and stored materials are responsible for the release of aldehydes that may interfere with embryonic development. There are also reports on the association between unhealthy environmental air conditions (bacteria, dust, particulate matter and volatile compounds) and reduction in the embryo formation rate and pregnancy.^16^ ^17^

Although air pollution has been associated with reproductive complications, the role that ambient air contaminants play on embryo cultures is poorly understood.^25^ ^26^ Recently, attention has been focused on VOCs and their role on in vitro human embryo cultures.^27^ ^28^ As the practices of IVF evolve toward the transfer of embryos at the blastocyst stage and the use of trophectoderm biopsy for preimplantation genetic testing (PGT), the extended period of in vitro culture required depends on a favorable and stable environment based on high-quality ambient air.^28^

A study conducted by Choe et al^29^ investigated the association between air pollutant levels and intrauterine pregnancy per cycle in women undergoing one or more in vitro fertilization procedures. This study has shown that exposure to VOCs during ovarian stimulation and embryo transfer is associated with decreased intrauterine pregnancy in IVF cycles.

The success of embryo implantation is also critically dependent on the environmental air conditions in the IVF laboratory.^30^ In the year 2000, researchers tested a laboratory prototype following regulations postulated by the National Environmental Balancing Bureau (NEBB) Procedural and Federal Standard 209E, which are in accordance with the Institute of Environmental Sciences and Technology (IEST) Recommended Practice (RP) 006.2,^12^ to meet the specific requirements of a Class 100/Class 10. The IVF Class-100/Class-10 laboratory clean room provides a laboratory environment with low particle count, virus-free, and with a low amount of bioaerosols, bacteria, mold spores, allergens and detectable volatile organic compounds. The researchers performed an electronic particle counting in air suspension and evaluated air velocity and uniformity, absolute filter pressure drop, water tightness and filter integrity. The tested clean room was free of VOCs, and it was isolated from environmental influences occurring within a large hospital. This environment mimics the in vivo conditions of human gametes and embryos. Therefore, these authors have demonstrated that the laboratory prototype is successful.^30^

Several studies^16^ ^17^ ^31^ investigated the effect of improved air quality on IVF and subsequent embryonic development after the construction of the clean room. They showed that clinical pregnancy rates decreased from 35% to 16% when numerous building odors were detected, and, after the construction of the clean room, they increased gradually, from 20% to 59%. Fertilization rates increased steadily after the construction of the clean room, from 62% to 69%. The proportion of embryos after the 4-cell stage was increased within 5 years after building the clean room, from 78% to 83%. According to these data, building a Class-100 clean room improved air quality and increased the number of embryos available for transfer after the 4-cell stage.

Heitmann et al^3^ evaluated specific parameters after infrastructural and management adjustments in the IVF laboratory. Air quality tests showed better air quality at the new IVF site. Embryo implantation (32.4% versus 24.3%, *p* < 0.01) and the number of live births (39.3% versus 31.8%, *p* < 0.05) increased significantly in the modified facility in comparison with the former one. More patients met the clinical criteria and were submitted to a single embryo transfer required on day 5, leading to a reduction in multiple gestation pregnancies. Improvements in laboratory conditions for IVF and air quality had profound positive effects on laboratory measurements and patient outcomes. The present study further reinforces the importance of the laboratory environment and air quality in the success of an IVF program.

Furthermore, in a study performed with 2,060 couples requesting IVF, the patients were treated in a clean room, and the outcome variables were compared with a cohort of 255 couples treated at a conventional facility.^32^ During the study period, birth rates increased (35.6% versus 25.8%, *p* = 0.02) and abortion rates decreased (28.7% versus 20.0%, *p* = 0.04) in the first 3 years after the construction of the clean room. Subsequently, the proportion of high-quality embryos increased steadily, whereas the pregnancy outcomes after intracytoplasmic sperm injection (ICSI) were sustained despite the increase in female age and decreased number of embryos transferred. This study demonstrates the feasibility of the manipulation of human gametes and embryo cultures in accordance with the Brazilian guidelines on air quality, and suggests that the performance of IVF in controlled environments can optimize their results.^32^

The implementation of good laboratory practices to improve IVF is of great interest to practitioners dealing with infertility. Structural modifications and installation of equipments, such as a VOC meter to measure gases that may interfere with pregnancy success rates, are highly desirable. A reduction in VOC concentration increases air quality, improving the rates of blastocyst formation, implantation, and clinical pregnancy. Due to fluctuations in air quality, it is necessary to optimize a laboratory methodology to improve the results of IVF.^33^

In the context of assisted reproduction techniques, air quality seems to have an impact mainly on follicular growth and embryogenesis.^34^ Subsequent basic studies are needed to better understand the systemic and cellular pathways through which air contaminants affect cell division and reproduction viability. The mechanism underlying the exposure to air pollution and the risk of pregnancy loss has not yet been completely understood. There probably is a potential longitudinal impact of the embryo culture environment on couples exposed to air pollution, which may lead to spontaneous abortion.^35^ In a prospective cohort of couples trying to conceive, the authors demonstrated that exposure to air contaminants throughout pregnancy was associated with pregnancy loss. The incidence of this outcome was 28% (*n* = 98) when additional domestic exposure to contaminants was considered.^36^

Since 1997, microbiological contamination in culture media have been routinely recorded, directly contributing to gestational outcomes in assisted fertilization.^37^ In a study performed with more than 13,000 cases in European human reproduction laboratories, an incidence of 0.67% of contamination in the culture plates was found.^38^ A similar prevalence study in Brazil found 4.8% contamination in the embryo plaques by bacteria and fungi, considering contamination as an important contributing factor of failure in assisted reproduction.^39^ Contamination may come from the air, from the equipment and from the materials used.^40^ Embryo culture medium plates are the best collection site to check for imminent microbiological contamination, as all of the various potential contaminants all affect them, which directly interfere with the rate of gestations and births.^41^

As there are no studies associating congenital diseases with embryonic contamination in assisted reproduction techniques, it is difficult to evaluate the comprehensiveness of contamination in public health. Even though it cannot be corroborated by studies in humans, there is evidence of gestational infections that impair the reproductive tract and cause malformation in bovine fetuses.^42^ The first observed consequence was a reduction in the formation of viable embryos for uterine transfer. The embryos may not survive the first cleavages, may present teratogenicity, or simply fail to implant in the uterus. Syndromes that compromise fetal health may also occur, bringing the possibility of increased stillbirths, prematurity, or birth of small concepts for gestational age, which was described in studies with cattle in which assisted fertilization was widely used.^42^ Although negative associations with air environment have been reported, little is known about the relevance of environmental microorganisms within human reproduction laboratories. In fact, most of the microorganisms isolated from a clean room environment are human commensals, and are probably irrelevant to reproductive cultures. In this regard, the use of measures such as the addition of antibiotics in culture media raises concerns as it may also cause damage and cytotoxic effects. With the reduction in microorganism density achieved through the construction of a clean room, further preventive measures are improved, such as low antibiotic levels needed within the culture medium.^43^

### Embryo Quality after Air Filtering System Replacement

Lack of laboratory standardization or recommendations based on particle control standards such as those in industrial clean rooms jeopardizes the definition of good practices for IVF laboratories. However, there is previous evidence that particle filtration alone improves embryo quality.^28^

In vitro fertilization laboratories should be equipped with HEPAs and activated carbon filters with positive pressure control to control airborne particles. There were significant differences in fertilization rates (83.7% versus 70.1%), embryo cleavage rate (97.35% versus 90.8%), blastocyst formation rate at day 5 (51.1% versus 41.7%), and pregnancy/implantation rates (54.6%, 34.4% versus 40.6%, 26.4%) after replacing the air filtration system.^44^

Kresowik et al^45^ analyzed 617 cycles of fertilization divided into 3 groups: before the removal of the filter, during its absence, and after its replacement. The following parameters were evaluated: fertilization; blastocyst formation; uterine embryo transfer day or cancellation; number of embryos transferred; implantation; pregnancy; and spontaneous abortion rates. The age of the patients was not different among the groups. Suboptimal air quality had a negative impact on fertilization and blastocyst formation. There was no significant difference observed in the implantation and pregnancy rates.

Comparing the fertilization rate and the rate of blastocyst formation in several studies,^21^ ^22^ ^23^ ^27^ we verified that there is a great difference between the use of incubators with VOC filtration and the use of incubators with HEPA filters. Higdon et al^27^ showed that the embryos cultured in an incubator with VOC filtration present 2.6 times more probability of developing blastocysts against those equipped with HEPA filtration. Munch et al^21^ reported that the cleavage rate was significantly affected in fresh cycles, but not in frozen cycles, suggesting that VOCs may negatively affect oocytes and zygotes preferentially. In the studies by Palter et al,^23^ there was a statistically significant increase in the blastocyst conversion rate, implantation rate and pregnancy rate, and a decrease in the pregnancy loss rate. Comprehensive control of chemical air components is critical to the success of preimplantation embryogenesis.

## Conclusion

Improvements in the environmental conditions and air quality of the IVF laboratory have profound positive effects on laboratory parameters and clinical outcomes. Replacing the air filtration system improves air quality by reducing the number of environmental particles (volatile organic compounds and microorganisms), and, thereby, improves fertilization rates, increases the number of blastocysts, the embryo implantation rates, the number of live births, and decreases the number of abortions. The evidence is clear that air quality affects the results of IVF. An increase in embryo quality and healthy children should always guide the assisted reproduction treatment. More controlled studies on air quality should be performed with the aim of investigating how particulate filtration interferes with IVF results and provides an effective balance of costs and benefits of replacing the air filtration system in the context of human assisted reproduction.
